# IRF5 Promotes Influenza Virus-Induced Inflammatory Responses in Human Induced Pluripotent Stem Cell-Derived Myeloid Cells and Murine Models

**DOI:** 10.1128/JVI.00121-20

**Published:** 2020-04-16

**Authors:** Jessica L. Forbester, Mathew Clement, Dannielle Wellington, Amy Yeung, Sandra Dimonte, Morgan Marsden, Lucy Chapman, Eve L. Coomber, Charlotte Tolley, Emily Lees, Christine Hale, Simon Clare, Irina Udalova, Tao Dong, Gordon Dougan, Ian R. Humphreys

**Affiliations:** a Division of Infection and Immunity/Systems Immunity, University Research Institute, Cardiff, United Kingdom; b MRC Human Immunology Unit, MRC Weatherall Institute of Molecular Medicine, University of Oxford, Oxford, United Kingdom; c Wellcome Trust Sanger Institute, Cambridge, United Kingdom; d Chinese Academy of Medical Sciences Oxford Institute, Nuffield Department of Medicine, Oxford University, Oxford, United Kingdom; e Department of Medicine, University of Cambridge, Cambridge, United Kingdom; f Kennedy Institute of Rheumatology, University of Oxford, Oxford, United Kingdom; University of North Carolina at Chapel Hill

**Keywords:** dendritic cells, IRF5, iPSCs, inflammation, influenza

## Abstract

Recognition of influenza A virus (IAV) by the innate immune system triggers pathways that restrict viral replication, activate innate immune cells, and regulate adaptive immunity. However, excessive innate immune activation can exaggerate disease. The pathways promoting excessive activation are incompletely understood, with limited experimental models to investigate the mechanisms driving influenza virus-induced inflammation in humans. Interferon regulatory factor 5 (IRF5) is a transcription factor that plays important roles in the induction of cytokines after viral sensing. In an *in vivo* model of IAV infection, IRF5 deficiency reduced IAV-driven immune pathology and associated inflammatory cytokine production, specifically reducing cytokine-producing myeloid cell populations in *Irf5*^−/−^ mice but not impacting type 1 interferon (IFN) production or virus replication. Using cytometry by time of flight (CyTOF), we identified that human lung IRF5 expression was highest in cells of the myeloid lineage. To investigate the role of IRF5 in mediating human inflammatory responses by myeloid cells to IAV, we employed human-induced pluripotent stem cells (hIPSCs) with biallelic mutations in *IRF5*, demonstrating for the first time that induced pluripotent stem cell-derived dendritic cells (iPS-DCs) with biallelic mutations can be used to investigate the regulation of human virus-induced immune responses. Using this technology, we reveal that IRF5 deficiency in human DCs, or macrophages, corresponded with reduced virus-induced inflammatory cytokine production, with IRF5 acting downstream of Toll-like receptor 7 (TLR7) and, possibly, retinoic acid-inducible gene I (RIG-I) after viral sensing. Thus, IRF5 acts as a regulator of myeloid cell inflammatory cytokine production during IAV infection in mice and humans and drives immune-mediated viral pathogenesis independently of type 1 IFN and virus replication.

**IMPORTANCE** The inflammatory response to influenza A virus (IAV) participates in infection control but contributes to disease severity. After viral detection, intracellular pathways are activated, initiating cytokine production, but these pathways are incompletely understood. We show that interferon regulatory factor 5 (IRF5) mediates IAV-induced inflammation and, in mice, drives pathology. This was independent of antiviral type 1 IFN and virus replication, implying that IRF5 could be specifically targeted to treat influenza virus-induced inflammation. We show for the first time that human iPSC technology can be exploited in genetic studies of virus-induced immune responses. Using this technology, we deleted IRF5 in human myeloid cells. These IRF5-deficient cells exhibited impaired influenza virus-induced cytokine production and revealed that IRF5 acts downstream of Toll-like receptor 7 and possibly retinoic acid-inducible gene I. Our data demonstrate the importance of IRF5 in influenza virus-induced inflammation, suggesting that genetic variation in the IRF5 gene may influence host susceptibility to viral diseases.

## Abstract (precis)

The inflammatory response to influenza A virus (IAV) participates in infection control but contributes to disease severity. After viral detection, intracellular pathways are activated, initiating cytokine production, but these pathways are incompletely understood. We show that interferon regulatory factor 5 (IRF5) mediates IAV-induced inflammation and, in mice, drives pathology. This was independent of antiviral type 1 IFN and virus replication, implying that IRF5 could be specifically targeted to treat influenza virus-induced inflammation. We show for the first time that human iPSC technology can be exploited in genetic studies of virus-induced immune responses. Using this technology, we deleted IRF5 in human myeloid cells. These IRF5-deficient cells exhibited impaired influenza virus-induced cytokine production and revealed that IRF5 acts downstream of Toll-like receptor 7 and possibly retinoic acid-inducible gene I. Our data demonstrate the importance of IRF5 in influenza virus-induced inflammation, suggesting that genetic variation in the IRF5 gene may influence host susceptibility to viral diseases.

## INTRODUCTION

During infection with influenza A virus (IAV), the host immune system must calibrate immune responses to control viral infection while minimizing damage to host tissues. Disease manifestations are often associated with the host inflammatory response to the virus (1, 2), and clinical outcomes vary widely between individuals (3). The inflammatory response is initiated when pattern recognition receptors (PRRs) on innate immune cells recognize IAV pathogen-associated molecular patterns (PAMPs), which trigger signaling cascades resulting in the expression of specific inflammatory cytokines and chemokines (4, 5). Cytokines play various roles, such as directly inhibiting viral replication and activating the cytolytic functions of T cells, whereas chemokines recruit innate immune cells such as macrophages, neutrophils, NK cells, and inflammatory monocytes to the lungs and airways (6).

Interferon regulatory factor 5 (IRF5) is a member of the IRF family of transcription factors, whose members have a shared N-terminal DNA binding domain and bind consensus interferon-stimulated response element (ISRE) motifs. As ISREs are enriched in the regulatory regions of immune genes, IRFs play key roles as master regulators in the innate immune response (7) and may provide a mechanism for conferring signal specificity to target gene sets downstream of Toll-like receptor (TLR) signaling (8). While IRF3 and IRF7 are necessary for the induction of type I interferons (9, 10), IRF5 has been shown to be key in regulating inflammatory cytokine responses, generally acting downstream of TLR-MyD88 pathways (11, 12). Genetic polymorphisms in the IRF5 gene in humans have been linked to various autoimmune conditions, including systemic lupus erythematosus, rheumatoid arthritis, Sjogren’s syndrome, multiple sclerosis, and inflammatory bowel disease (13). IRF5 has also been shown to be important in regulating immune responses to various pathogens in murine and human cell models (14–17). Additionally, *Irf5*^−/−^ mice are resistant to systemic shock induced by CpG ligands and lipopolysaccharide (LPS) (12). The extent to which IRF5 contributes to inflammation-induced pathologies, however, is unclear.

IRF5 is expressed predominantly by myeloid cells such as dendritic cells (DCs) and macrophages, in addition to B cells (13, 18). Myeloid cells can have protective and immunopathogenic roles during IAV infection, producing inflammatory cytokines and initiating adaptive immune responses (19). Furthermore, inflammatory monocytes and monocyte-derived DCs have been identified to drive inflammation and lung pathology during IAV infection (19, 20).

Studying inflammatory cytokine responses in human myeloid cells is challenging. Human DCs are difficult to culture *in vitro* and, although DCs can be induced from blood-derived monocytes, these cells display morphological and functional differences from human primary DCs, for example, differing in their capacities for T cell stimulation in comparison to CD11c^+^ blood-derived DCs (21). Primary myeloid cells are also difficult to genetically manipulate, meaning that studies addressing the effect of host genetics on myeloid cell responses can be challenging. Human induced pluripotent stem cells (hIPSCs) offer a useful system for studying host-pathogen variations because these cells are amenable to genetic manipulation, can be differentiated toward multiple cellular lineages, and are self-renewing, allowing for the production of sufficient quantities of cells of the same genetic background. hIPSC-derived macrophages (iPSDMs) have already been used to successfully model the interactions of pathogens with host cells (16, 22). However, to date, hIPSC technology has not been used to perform genetic investigations of virus-induced immune responses. To study the impact of IRF5 on human myeloid IAV-induced immune responses, we utilized hIPSCs generated from a healthy donor or with mutations in *IRF5* generated by CRISPR-Cas9 engineering differentiated into dendritic cells and macrophages as a human model system to assess the role of IRF5 in the regulation of immune responses to IAV. Using these tools in combination with studies of human lung cells, in addition to *Irf5^−/−^* mice, we show that IRF5 drives IAV-induced inflammatory cytokine responses in mice and humans without impacting virus replication and type 1 interferon (IFN) secretion, and this process mediates viral pathogenesis *in vivo*.

## RESULTS

### IRF5 mediates inflammatory cytokine and myeloid cell responses to influenza A virus infection in mice.

**FIG 1:**
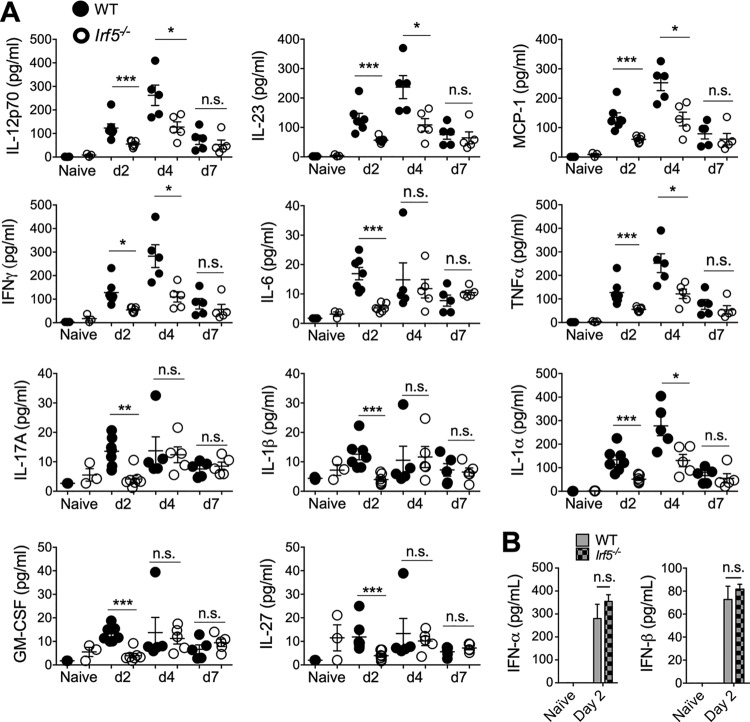
IRF5 alters cytokine responses to influenza A virus in a murine infection model. WT and *Irf5*^−/−^ mice were infected intranasally with 3 × 10^3^ A/X-31 influenza virus. (A) Inflammatory cytokine expression in BAL fluid was measured using multiplex assays 2, 4, and 7 days p.i. Data shown are the mean ± SEM using 7 WT and 5 *Irf5*^−/−^ mice (day 2) or five mice per genotype (day 4 and day 7) and represent the results from two independent experiments. (B) IFN-α and IFN-β levels in BAL fluid measured by ELISA in *Irf5^−/−^* and WT naive and IAV-infected mice at 2 days p.i. Data shown are the mean ± SEM of the results from 3 to 6 mice per group at 2 days p.i.

The mouse is the primary experimental model for studying the immunological response to IAV, where it has been demonstrated that excessive inflammatory cytokine and cellular immune responses promote lung pathology (2, 23, 24). We first used this model to assess whether IRF5 impacts influenza virus-induced immune responses during IAV infection *in vivo*, using the low-pathogenicity murine-adapted H3N2 influenza A virus (A/X-31). Prior studies have indicated that viral infections of *Irf5^−/−^* mice lead to reduced cytokine production in comparison to wild-type (WT) controls (14, 17, 25). In accordance, we observed a significant reduction in early cytokine release in *Irf5^−/−^* mice, with interleukin 23 (IL-23), IFN-γ, tumor necrosis factor alpha (TNF-α), methyl-accepting chemotaxis protein 1 (MCP-1), IL-6, IL-17A, IL-1α, IL-12p70, granulocyte-macrophage colony-stimulating factor (GM-CSF), IL-1β, and IL-27 all significantly reduced in the bronchoalveolar lavage (BAL) fluid of *Irf5^−/−^* mice in comparison to WT controls 2 days postinfection (p.i.) (Fig. 1A), with some cytokines remaining significantly reduced in *Irf5^−/−^* mice 4 days p.i. (Fig. 1A). In contrast to other viral infections (17), IFN-α or IFN-β production in response to influenza infection was unaltered (Fig. 1B) at a time point (day 2 p.i.) previously demonstrated to represent the time of significant A/X-31 influenza virus-induced type 1 IFN secretion in this model (26). These data therefore imply that IRF5 selectively modulates the expression of certain influenza virus-induced inflammatory cytokines independently of type I IFNs in mice.

**FIG 2:**
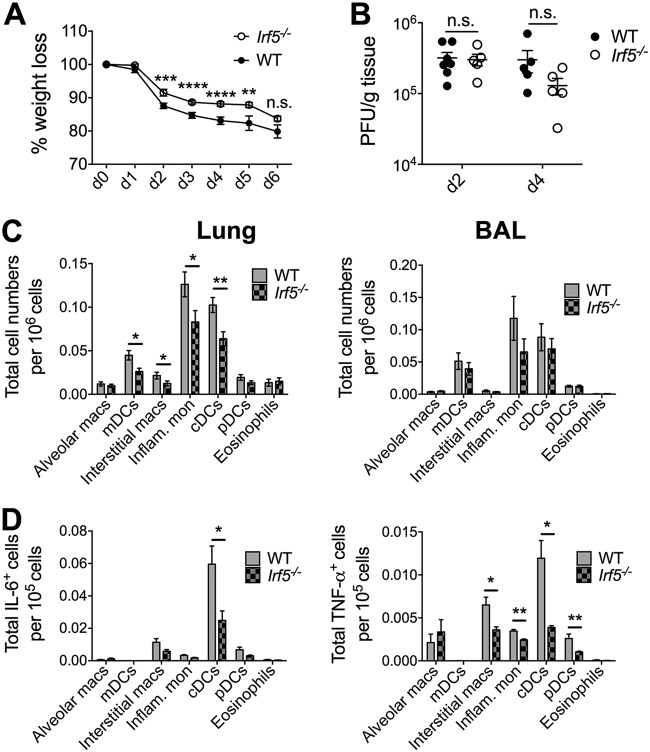
IRF5 enhances influenza A virus-induced inflammatory response in a murine infection model. (A) Weight loss of WT and *Irf5^−/−^* mice was assessed over time, and comparable results were observed in 4 independent experiments, with 4 to 5 WT or *Irf5*^−/−^ mice in each group per experiment. Data shown are the mean ± SEM. (B) Replication of virus in the lungs was quantified using a plaque assay. Data shown are the mean ± SEM using 7 WT and 5 *Irf5*^−/−^ mice for day 2 and 5 mice of each genotype for day 4. (C) Recruitment of specific myeloid cell populations (mDCs, monocyte-derived DCs; cDCs, conventional DCs; pDCs, plasmacytoid DCs; Inflam. mon, inflammatory monocytes) in WT and *Irf5*^−/−^ mice was assessed by flow cytometry 2 days p.i. Populations were defined by the following markers: alveolar macrophages (Alveolar macs), SiglecF^+^ CD11b^+^ CD64^+^ Ly6C^−^; mDCs, SiglecF^−^ CD11b^+^ MHC-II^+^ CD11c^+^ CD64^+^ Ly6C^+^; interstitial macrophages, SiglecF^−^ CD11b^+^ MHC-II^+^ CD11c^−^ CD64^+^ Ly6C^+^; inflammatory monocytes, SiglecF^−^ CD11b^+^ MHC-II^−^ Ly6C^+^ CD64^+^; cDCs, MHC-II^+^ CD11c^+^ Ly6C^−^; pDCs, B220^+^ SiglecH^+^ MHC-II^low^ CD11c^low^; and eosinophils, SiglecF^+^ CD11c^−^ CD11b^+^ Ly6C^−^. Data shown are the mean ± SEM using 11 WT and 10 *Irf5^−/−^* mice from multiple replicates. (D) The total number of each individual myeloid cell population (unstimulated, *ex vivo*) positive for IL-6 and TNF-α expression was detected by flow cytometry, with data presented representing the mean total cell number per 10^5^ cells of each cell type ± SEM. Data represent two experiments.

Early reduction in inflammatory cytokine production in *Irf5^−/−^* mice was accompanied by a moderate amelioration of IAV-induced weight loss (Fig. 2A). Interestingly, a recent study reported that reduced IAV-induced cytokine production in *Irf5^−/−^* mice was associated with reduced virus replication (25). However, at a time point where we observed substantially reduced cytokine production (day 2 p.i.), we observed no alteration in IAV load in *Irf5*^−/−^ mice (Fig. 2B), nor did we observe altered virus load in *Irf5^−/−^* mice at a later time point of 4 days p.i. (Fig. 2B). Thus, our data demonstrate for the first time that IRF5 promotes IAV-induced weight loss independently of an impact on influenza virus replication.

Given the established role for myeloid cells in pulmonary inflammation during IAV infection (27, 28), we used polychromatic flow cytometry panels to assess whether Irf5 influenced myeloid cell accumulation during infection. Reductions in monocyte-derived DCs, interstitial macrophages, inflammatory monocytes, and conventional DCs in the lungs of *Irf5^−/−^* mice were observed at 2 days p.i. (Fig. 2C). Importantly, lower cytokine responses in *Irf5^−/−^* mice were accompanied by significant reductions in IL-6^+^ cDCs and TNF-α^+^ cDCs, interstitial macrophages, and cDCs and pDCs in the airways (Fig. 2D). Thus, these data suggested that Irf5 plays a key role in shaping the early innate inflammatory response during influenza virus infection and point to a central role for myeloid cells in promoting IRF5-driven viral disease.

### Myeloid cells in the human lung express high levels of IRF5.

**FIG 3:**
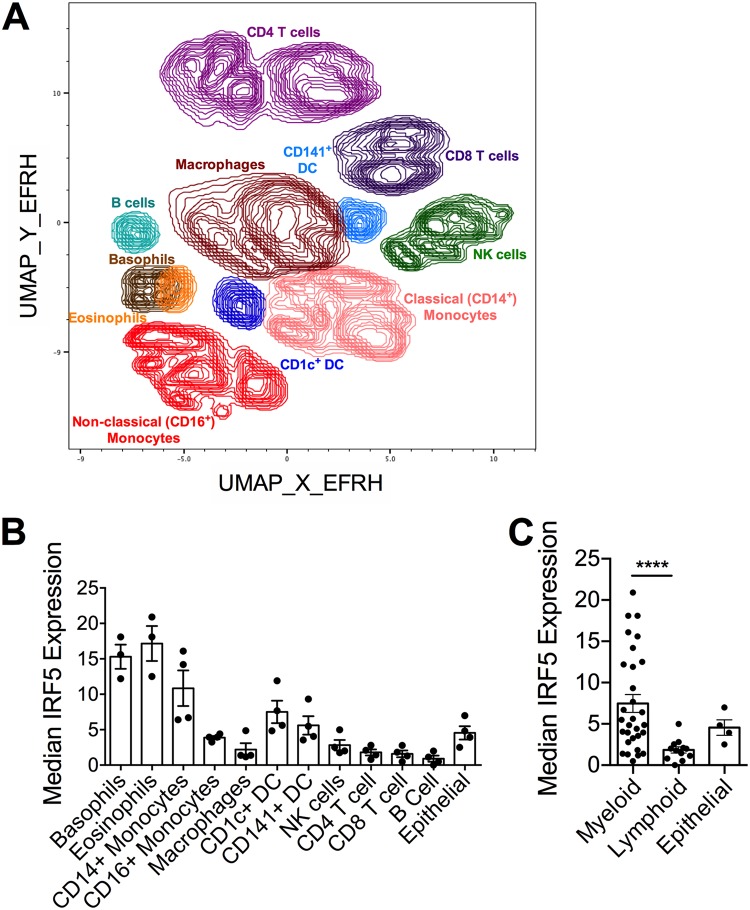
IRF5 expression in human lung cells. IRF5 expression by multiple cellular subsets derived from human lung tissue from independent donors was analyzed by CyTOF. (A) UMAP based on downsampled, concatenated files from lung samples from four donors using phenotypic markers. Post-UMAP analysis, populations (colored by cell type as identified by lung CyTOF) were defined via the following markers: CD4^+^ T cells, CD3^+^ CD4^+^ CD20^−^; CD8^+^ T cells, CD3^+^ CD20^−^ CD8^+^; B cells, CD3^−^ CD20^+^; NK cells, CD3^−^ CD20^−^ CD56^+^; CD14^+^ monocytes, CD16^−^ CD11b^+^ CD14^+^ HLA-DR^+^; CD16^+^ monocytes, CD14^−^ CD11b^+^ CD16^+^ HLA-DR^+^; macrophages, CD11b^+^ CD68^+^ HLA-DR^+^; pDCs, CD123^+^ CD11b^+^ HLA-DR^+^; CD141^+^ cDCs, CD11b^+^ HLA-DR^+^ CD1c^−^ CD141^+^; CD1c^+^ cDCs, CD11b^+^ HLA-DR^+^ CD1c^+^ CD141^−^; eosinophils, Siglec8^+^ CD123^−^; and basophils, Siglec8^+^ CD123^+^. (B) Median IRF5 expression in populations identified in panel A from lung samples taken from four independent donors, corrected for nonspecific staining using unpermeabilized controls for each sample, and error bars represent the SEM. (C) Median IRF5 expression in myeloid versus lymphoid cell subsets, and error bars represent the SEM.

Although the mouse is a useful model for probing immune responses to IAV, numerous differences exist between the mouse and human immune systems. It was therefore important to investigate the role of human IRF5 in IAV-induced immune responses. We first measured IRF5 expression in multiple cell subsets in human lung samples using CyTOF (Fig. 3A). Using lung samples from four independent donors, we identified significantly different IRF5 expression levels dependent on cell subset (*P* = 0.0252, *R*^2^ = 0.9098) (Fig. 3B), with cells of the myeloid lineage, particularly eosinophils, basophils, and monocytes, displaying the highest expression of IRF5 in the human lung. Expression was higher in CD1c^+^ DCs and CD141^+^ DCs than in lung resident macrophages, where expression was relatively low. Furthermore, when IRF5 expression data were combined for all myeloid cell subsets and all lymphoid cell subsets (Fig. 3C), the expression of IRF5 was significantly higher in myeloid cells than in lymphoid cells (median expression of myeloid cells, 6.01; median expression of lymphoid cells, 1.79; *P* < 0.0001), suggesting that IRF5 expression is highest in the myeloid compartment.

### iPSCs with a biallelic mutation in *IRF5* can be differentiated into conventional dendritic cells.

**FIG 4:**
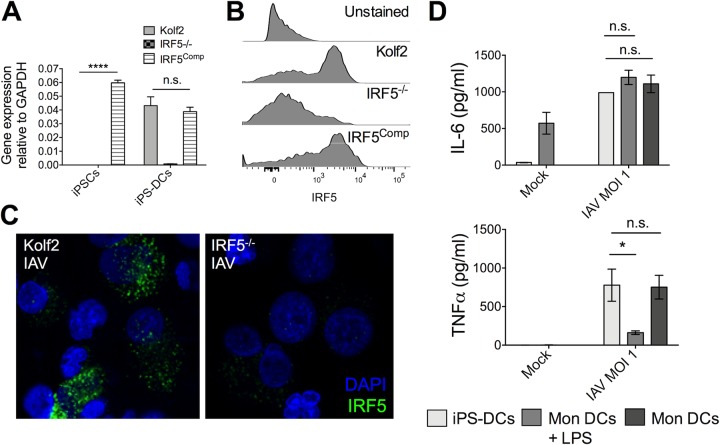
*IRF5*^−/−^ iPSCs, IRF5^Comp^ iPSCs, and Kolf2 iPSCs can be differentiated into iPS-DCs which lack or express IRF5. CRISPR-Cas9 was used to generate biallelic mutations in *IRF5* in the Kolf2 background. IRF5^Comp^ iPSCs were generated using TALEN-mediated integration of *IRF5* into the IRF5^−/−^ background. (A) Relative expression of *IRF5* in iPSCs and iPS-DCs relative to *GAPDH*. Data are shown as four technical replicates per assay, with assays repeated three times from independent iPS-DC batches. (B) Flow cytometry showing IRF5 expression in iPS-DCs generated from IRF5^−/−^, IRF5^Comp^, and Kolf2 iPSCs. (C) Immunostaining for IRF5 in A/X-31 influenza (IAV)-infected Kolf2 and IRF5^−/−^ iPS-DCs (DAPI, blue; IRF5, green). (D) IL-6 and TNF-α production 24 h p.i. by IAV-challenged Kolf2 iPS-DCs and monocyte-derived DCs generated from human peripheral blood, either with or without 48 h LPS maturation, were assayed by ELISA. Data represented show the mean ± SEM from three independent Kolf2 differentiations for iPS-DCs, and from three independent healthy donors for monocyte-derived DCs.

Given that, in the human lung, IRF5 expression was highest in cells of the myeloid lineage and that in mice Irf5 promoted proinflammatory cytokine production by myeloid cells in response to IAV infection, we next sought to establish a human myeloid cell model to scrutinize the role of IRF5 in the myeloid cell cytokine response to IAV. We differentiated an hIPSC line with a biallelic mutation in IRF5 generated using CRISPR-Cas9 genome editing (16) and the parental line Kolf2 into iPS-DCs using a published differentiation strategy (29). We also generated a complemented isogenic control line for IRF5^−/−^ (here, IRF5^Comp^) to confirm the IRF5 dependency of any phenotypes observed (30). To confirm gene editing strategies, we examined the expression of IRF5 in Kolf2, IRF5^−/−^, and IRF5^Comp^ iPS-DCs. *IRF5* mRNA was detected in Kolf2 iPS-DCs but not IRF5^−/−^ iPS-DCs, with expression restored in IRF5^Comp^ iPS-DCs (Fig. 4A). Similar restoration of IRF5 protein expression in IRF5^Comp^ iPS-DCs was observed (Fig. 4B). Furthermore, after infection of iPS-DCs with IAV, IRF5 was detected in Kolf2 iPS-DCs and not IRF5^−/−^ iPS-DCs by immunostaining (Fig. 4C). We then compared IAV-induced cytokine production by iPS-DCs from our healthy control iPSC line Kolf2 with monocyte-derived DCs from the blood of three healthy donors which were left either immature or matured for 48 h with LPS. iPS-DCs demonstrated cytokine profiles after IAV infection similar to those of immature monocyte-derived DCs (Fig. 4D), validating iPS-DCs as an experimental system for examining virus-induced cytokine responses.

**FIG 5:**
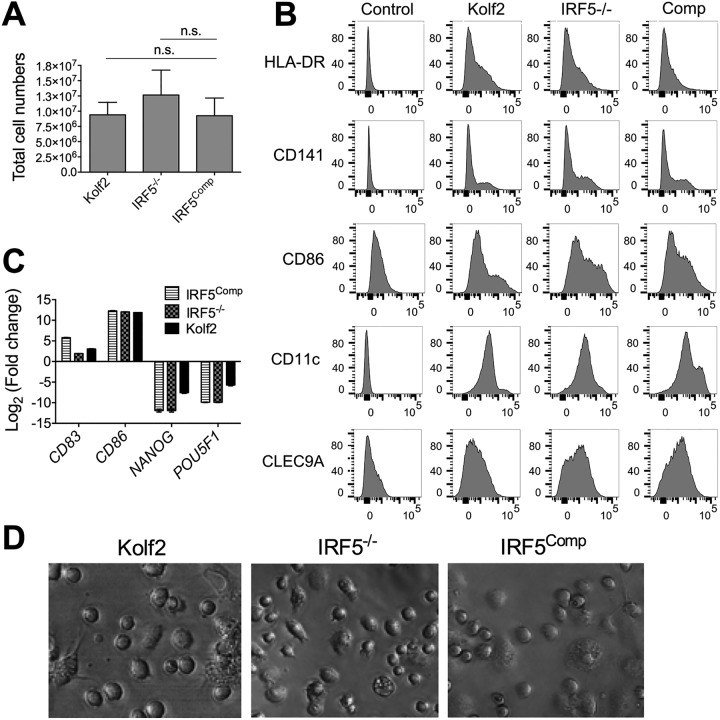
*IRF5*^−/−^ iPSCs, IRF5^Comp^ iPSCs, and Kolf2 iPSCs can be differentiated into iPS-DCs that display similar morphologies. *IRF5*^−/−^ iPSCs, IRF5^Comp^ iPSCs, and Kolf2 iPSCs were differentiated into dendritic cells using defined concentrations of growth factors to generate embryoid bodies (EBs) and GM-CSF and IL-4 to generate immature DCs from these EBs. (A) Total cell numbers of DC precursors harvested from DC differentiation plates. Data shown are from 8 independent differentiations per iPSC line. (B) Surface expression of DC markers was examined via flow cytometry in Kolf2, IRF5^−/−^, and IRF5^Comp^ iPS-DCs. Representative plots are presented from one experiment, with experiments performed at least three times. (C) Gene expression of DC markers *CD83* and *CD86* and iPSC markers *NANOG* and *POU5F1* by iPS-DCs relative to *GAPDH* was quantified using TaqMan gene expression assays. The data shown represent four technical replicates per assay, with assays repeated at least twice from independent iPS-DC batches. (D) Morphologies of iPS-DCs generated from Kolf2, IRF5^Comp^, and IRF5^−/−^ iPSCs.

It has previously been shown that IRF5 deficiency or transcription activator-like effector nuclease (TALEN)-based engineering targeting the adeno-associated virus integration site 1 (AAVS1) does not affect the differentiation capacity of iPSCs to iPS-derived macrophages (iPSDMs) (16, 31). To ensure that genome editing strategies had not altered the differentiation capacity of iPSCs to dendritic cells, we assessed the differentiation efficiencies of Kolf2, IRF5^−/−^, and IRF5^Comp^ iPSCs. We observed similar numbers of cells harvested from embryoid bodies (EBs) from days 19 to 24 of differentiation, with no significant difference in the numbers of cells harvested from eight independent differentiations per line (Fig. 5A). After the completion of the 25-day DC differentiation, DC marker expression was examined by flow cytometry, with CD141, CLEC9A, CD11c, major histocompatibility complex class II (MHC-II), and CD86 similarly expressed in all three iPS-DC lines (Fig. 5B). There are three main subsets of human DCs, pDCs and two subsets of myeloid (conventional) DCs, CD1c^+^ and CD141^+^, with DC hematopoiesis distinct from the development of monocytes (32). iPS-DCs express markers of human conventional DCs, including CD11c and CD141 (Fig. 5B), as well as HLA-DR, CD86, and CLEC9A, which have been shown to be expressed by human CD141^+^ DCs (33). We did not detect CD303 expression, a marker for pDCs, or CD1c, the marker for the other subset of human conventional dendritic cells (34). As observed by Sachamitr et al. (29), we also detected CD14 and dendritic cell-specific intercellular adhesion molecule-3-grabbing nonintegrin (DC-SIGN) expression by iPS-DCs (J. L. Forbester and I. R. Humphreys, unpublished data). Gene expression analysis confirmed similar induction of the DC markers *CD83* and *CD86* in all three iPSC lines after differentiation to iPS-DCs and the loss of expression of the pluripotency markers *NANOG* and *POU5F1* (Fig. 5C). The morphologies of Kolf2, IRF5^−/−^, and IRF5^Comp^ iPS-DCs in culture were indistinguishable (Fig. 5D). Taken together, these data suggest that neither IRF5 deficiency nor TALEN-based engineering influences iPSC differentiation.

### IRF5 enhances IAV-induced inflammatory cytokine production in iPS-DCs.

After confirming that IRF5 deficiency did not alter iPS-DC surface phenotype or morphology (Fig. 5) and that iPS-DCs exhibit cytokine profiles similar to those of human monocyte-derived DCs after IAV stimulation (Fig. 4D), we next used iPS-DCs to determine whether IRF5 has a cell-intrinsic role in human DC cytokine production, in particular, the proinflammatory cytokines IL-6 and TNF-α. Despite a protective role for IL-6 being reported in murine models of IAV infection (35, 36), high production of IL-6 is linked to the severity of symptoms in human patient cohorts (37, 38), whereas TNF-α has been shown to enhance cellular inflammation and pathology during IAV infection (23).

**FIG 6:**
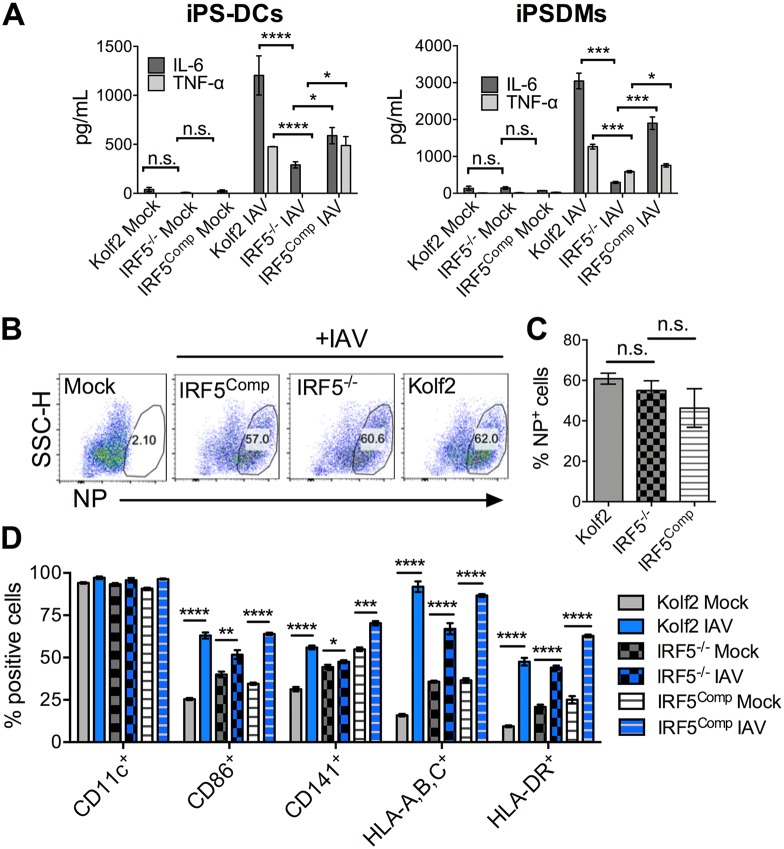
IRF5 enhances IAV-induced inflammatory cytokine production in iPS-DCs and iPSDMs. (A) IL-6 and TNF-α were measured by ELISA in supernatants harvested from iPS-DCs and iPSDMs generated from an iPSC line with a biallelic mutation in *IRF5*, compared to the parent line Kolf2, and a line with a functional *IRF5* gene was reintroduced into the AAVS1 integration site by TALEN engineering after infection with IAV at an MOI of 1. Supernatants were harvested at 24 h for assays; data shown represent the mean ± SEM for triplicate wells from at least 3 independent experiments. (B) IRF5^−/−^, IRF5^Comp^, and Kolf2 iPS-DCs were infected with IAV at an MOI of 1 and then stained for IAV NP 24 h postinfection and analyzed via flow cytometry. SSC-H, side scatter height. (C) Percentage of positive NP iPS-DCs 24 h postinfection with IAV, with data presented showing the mean ± SEM from three independent experiments. (D) Expression of DC maturation surface markers for iPS-DCs generated from IRF5^−/−^, Kolf2, or IRF5^Comp^ hIPSCs 24 h postinfection with A/X-31 influenza (IAV) at an MOI of 1, as measured by flow cytometry, with data presented showing the mean ± SEM from three independent experiments.

Twenty-four hours after stimulation of iPS-DCs with IAV, IL-6 and TNF-α production by IRF5^−/−^ iPS-DCs was significantly reduced in comparison to that in Kolf2 iPS-DCs, whereas cytokine production was restored in IRF5^Comp^ iPS-DCs (Fig. 6A, iPS-DCs). IRF5 deficiency had no impact on virus entry, as indicated by comparable influenza nucleoprotein (NP) staining after 24 h (Fig. 6B and C). In addition, there was no significant difference in cell viability after IAV stimulation, as measured by live viability dye and flow cytometry 24 h p.i. (mean ± standard error of the mean [SEM] percent of live cells: 65.767% ± 0.353% for IRF5^Comp^, 63.95% ± 1.655% for IRF5^−/−^, and 70% ± 1.654% for Kolf2). Surface analysis of DC markers by flow cytometry showed that the number of iPS-DCs expressing DC maturation markers after IAV infection was similarly significantly increased in Kolf2, IRF5^−/−^, and IRF5^Comp^ iPS-DCs (Fig. 6D). Moreover, gene complementation has previously been used to inhibit immune responsiveness in the context of restoration of expression of the inhibitory IL-10 receptor into *IL10RB^−/−^* iPS-derived myeloid cells (31). Thus, we do not believe that the restored cytokine responsivess of complemented IRF5^−/−^ cells is a consequence of nonspecific induction of cytokine secretion by the complementation process but instead is due to IRF5 itself. Collectively, these data suggest that IRF5 deficiency selectively alters iPS-DC cytokine production after exposure to IAV. In addition, to probe IRF5 deficiency in a different myeloid cell lineage, we differentiated IRF5^−/−^, Kolf2, and IRF5^Comp^ iPSCs to macrophages (iPSDMs) using a slightly modified version of a previously published protocol (22), demonstrating a significant reduction in IL-6 and TNF-α production similar to that observed in iPS-DCs (Fig. 6A, iPSDMs).

### IRF5 acts downstream of TLR7 and, possibly, RIG-I signaling pathways to drive human myeloid cell cytokine responses to IAV.

**FIG 7:**
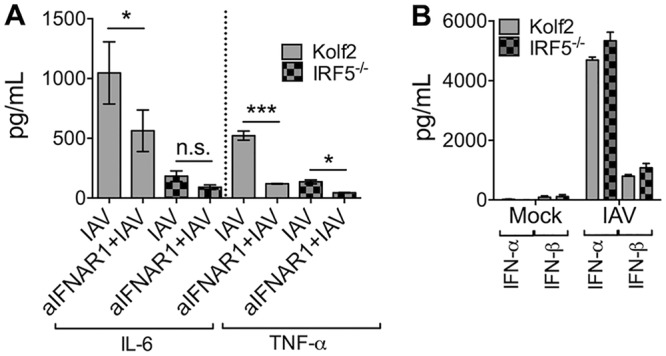
Type I IFN signaling enhances IL-6 and TNF-α production by iPS-DCs. A total of 2 × 10^4^ iPS-DCs were challenged as stated below for each assay, and supernatants were harvested after 24 h, unless otherwise stated. A/X-31 influenza (IAV) was used at an MOI of 1. (A) Cells were preincubated for 1 h with anti-IFNAR1 antibody or left untreated prior to viral infection. Data shown represent the mean ±SEM for triplicate wells from at least 3 experiments. Supernatants were harvested and assayed for IL-6 and TNF-α by ELISA. (B) Supernatants from mock or IAV-infected Kolf2 or IRF5^−/−^ iPS-DCs were harvested at 24 h and assayed for IFN-α and IFN-β by ELISA. Data shown represent 2 separate experiments.

In some experimental systems, IRF5 mediates virus-induced production of type I IFN (17, 39). Given that type I IFN is implicated in driving influenza virus-induced inflammatory cytokine responses (26), we assessed whether IRF5 deficiency impacted influenza virus-induced IFN production. Blocking type I IFN reduced IAV-induced IL-6 and TNF-α production, albeit, in the case of IL-6 not to levels produced by IRF5^−/−^ iPS-DCs (Fig. 7A). Furthermore, IRF5 had no impact on type I IFN secretion by iPS-DCs (Fig. 7B). Thus, although type I IFN enhanced IRF5-induced proinflammatory cytokine secretion, the production of type I IFN by iPS-DCs was not an IRF5-regulated process.

**FIG 8:**
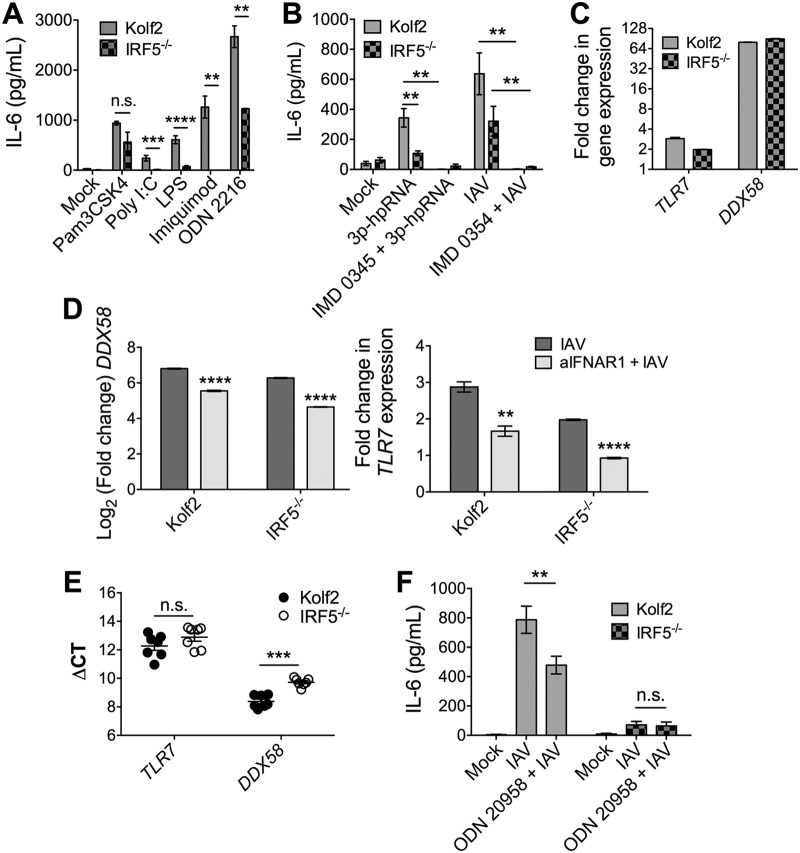
IRF5 acts downstream of TLR7 and RIG-I to drive inflammatory cytokine responses in iPS-DCs. A total of 2 × 10^4^ iPS-DCs were challenged as stated below for each condition in each assay, and supernatants were harvested after 24 h. A/X-31 influenza virus (IAV) was used at an MOI of 1. For blocking assays, cells were either preincubated for 1 h with inhibitor (IMD 0354, IKKβ inhibitor), or inhibitor was added directly with viral inoculum (ODN 20958, TLR7 inhibitor). Data shown represent the mean ± SEM of the results for triplicate wells from at least 3 experiments, unless otherwise stated. (A) IL-6 production by Kolf2 and IRF5^−/−^ iPS-DCs in response to stimulation with various TLR ligands (TLR2, Pam3CSK4, 300 ng/ml; TLR3, poly(I·C), 50 μg/ml; TLR4, LPS, 50 μg/ml; TLR7, imiquimod, 50 μg/ml; TLR9, ODN 2216, 3 μg/ml) was measured by ELISA. Data shown represent four wells per condition for one iPS-DC batch per line, with assays replicated in two independent experiments. (B) IL-6 response as measured by ELISA in Kolf2 and IRF5^−/−^ iPS-DCs to RIG-I ligand 3p-hpRNA with or without IKKβ inhibitor IMD 0354 and to IAV with or without IMD 0354. (C) Fold change in mRNA levels for *TLR7* and *DDX58*, measured by RT-qPCR using *GAPDH* as an endogenous control. (D) *DDX58* and *TLR7* mRNA levels in iPS-DCs after IAV infection with or without blocking of type I IFN signaling using anti-IFNAR1. Data shown represent four technical replicates per assay, with assays repeated at least twice from independent iPS-DC batches. (E) Relative mRNA levels of *TLR7* in iPS-DCs generated from IRF5^−/−^ iPSCs or parent Kolf2 iPSCs, measured using RT-qPCR. (F) IL-6 response as measured by ELISA in Kolf2 and IRF5^−/−^ iPS-DCs to A/X-31 influenza virus with or without TLR7 inhibitor ODN 20958.

We next investigated which PRRs require IRF5 to elicit cytokine responses following IAV stimulation of iPS-DCs. IAV is detected by endosomal TLR7 and members of the DExDC helicase family and RIG-I in dendritic cells (40–42). Consistent with data from human and murine macrophages (12, 43), IRF5^−/−^ iPS-DCs produced significantly less IL-6 in response to agonists of TLR7 (and TLRs 4, 3, and 9, Fig. 8A). Moreover, stimulation of IRF5^−/−^ iPS-DCs with the RIG-I-specific agonist 3p-hpRNA led to a significant reduction in IL-6 production (Fig. 8B), demonstrating that IRF5 mediates RIG-I- and TLR7-induced responses in iPS-DCs. Substantial IFN-dependent induction of the RIG-I-encoding gene *DDX58* and, to a lesser extent, *TLR7*, was observed upon IAV stimulation of iPS-DCs (Fig. 8C and D), and IRF5 deficiency did not impair baseline PRR expression (Fig. 8E), suggesting that the reduced expression of proinflammatory cytokines in response to TLR7, RIG-I, and IAV stimulation in IRF5^−/−^ iPS-DCs was not a consequence of restricted PRR expression by these cells.

Finally, we wanted to determine which PRRs mediated IAV-induced cytokine responses in iPS-DCs. Because (i) there is no selective antagonist of RIG-I and (ii) human CD141^+^ DCs express TLR7 (44), we focused on the role of TLR7 in mediating IAV-induced iPS-DC cytokine responses. The addition of the specific TLR7 antagonist ODN 20958 to IAV-stimulated Kolf2 iPS-DCs significantly abated IL-6 production, whereas TLR7 inhibition in IRF5^−/−^ iPS-DCs did not further reduce IAV-induced IL-6 responses (Fig. 8F). These data suggest that IRF5 promotes TLR7-mediated cytokine responses following IAV detection by human DCs. However, IAV-induced cytokine secretion was incompletely inhibited by TLR7 blockade in Kolf2 iPS-DCs, suggesting that other PRRs contribute to IRF5-mediated responses. IκB kinase beta (IKKβ) has been shown to play a crucial role in IRF5 and NF-κB activation (11). In support of this, pretreatment of IAV-stimulated iPC-DCs with the IKKβ inhibitor IMD 0354 dramatically reduced IAV-induced cytokine production by iPS-DCs (Fig. 8B), implying that other PRRs, including (possibly) RIG-I, contribute to IAV-induced cytokine responses by iPS-DCs in addition to TLR7.

## DISCUSSION

Here, using iPS-DCs as a model system, we have shown that IRF5 expression by myeloid cells is important in driving the inflammatory response to IAV without impacting viral uptake by iPS-DCs or DC maturation. Using various blocking assays and stimulation with TLR/RIG-I-like receptor (TLR/RLR) ligands, we show that IRF5 is most likely acting downstream of TLR7 and, possibly, RIG-I signaling to drive the production of proinflammatory cytokines.

Given that IFN-stimulated genes contribute to anti-influenza immunity (5), to identify whether IFR5 and/or related pathways could be safely exploited to dampen inflammatory cytokine responses to influenza virus, it is important to understand the relationship between IRF5 and virus-induced type 1 IFN. We found that IRF5^−/−^ iPS-DCs and *Irf5^−/−^* mice were not deficient in type I IFN production but that type I IFN enhances the IRF5-mediated inflammatory cytokine response, a process associated with IFN-mediated induction of TLR7 and RIG-I. Although certain studies have reported a role for IRF5 in type I IFN induction directly in certain contexts (25, 43, 45), functional redundancies between IRF proteins may exist (46). Also, although a role for IRF5 in promoting type 1 IFN secretion following influenza virus infection *in vivo* has been reported, the same studies observed reduced virus replication in *Irf5*^−/−^ mice, precluding the possibility of uncoupling decreased virus replication and subsequent pattern recognition receptor stimulation from a direct influence of IRF5 on type 1 IFN expression. Furthermore, early *in vivo* studies of Irf5 responses to viruses may have been confounded by a *Dock2* mutation prevalent in *Irf5^−/−^* mouse colonies (46). Also, it has been demonstrated *in vitro* that unlike IRF3 and IRF7, IRF5 does not bind to the virus-response elements in IFN promoters (47). IRF5-mediated induction of type I IFN may also be virus specific, at least *in vitro*, with Newcastle disease virus (NDV), vesicular stomatitis virus (VSV), and herpes simplex virus 1 (HSV-1) infection shown to activate IRF5 but lead to the induction of distinct *IFNA* gene subtypes in human cell lines (48). IRF3 and IRF7 are activated by IAV, and these transcription factors have been shown to be necessary for inducing type I IFN after IAV infection (49, 50). Therefore, we suggest that after sensing of IAV by dendritic cells, IRF3, IRF5, and IRF7 are induced, resulting in the production of type I IFN and inflammatory cytokines, with the type I IFN induced by IRF3 and IRF7 exacerbating IRF5 activation, likely in part through the induction of TLR7 and, possibly, RIG-I expression. In the context of influenza virus pathogenesis, our data imply that IRF5 could be safely targeted to limit virus-induced proinflammatory cytokine production without affecting IFN production and the associated induction of antiviral effector genes.

The phenotype of reduced inflammatory cytokines observed in our iPS-DC model was also evident *in vivo* using *Irf5^−/−^* mice. Although we observed no mortality in either WT or *Irf5^−/−^* mice (J. L. Forbester and I. R. Humphreys, unpublished data), we observed reduced cytokine production by myeloid cells that correlated with reduced cellular pulmonary infiltration and a moderate impact on virus-induced weight loss. Although *Irf5^−/−^* mice have previously been shown to be less susceptible to IAV-induced pathology (25), in this study, we were able to decouple viral load and inflammatory cytokines in the early stages of infection, demonstrating that the enhanced pathology in WT compared to *Irf5^−/−^* mice was immune-mediated rather than a consequence of heightened virus replication. Why differences exist between our data and those obtained by Chen et al. (25) is unclear, although this may reflect the different influenza virus strains (H3N2 versus H1N1) used in these experiments. Irrespective, the data presented herein demonstrate that IRF5 modulates viral pathogenesis via the regulation of inflammation and not virus replication, and that targeting this pathway as an adjunct therapy to antiviral drug treatment may represent an effective therapeutic approach to the treatment of influenza virus pathogenesis.

Although the mouse provides a useful model to study viral pathogenesis, there are inherent immunological differences between mice and humans (51). Therefore, we wanted to establish a human cell system amenable to genetic manipulation, so that gene function can be understood in the context of a human cellular environment. Given that primary human myeloid cells are difficult to genetically manipulate and access in large numbers, hIPSCs offer a solution; once hIPSCs are generated, they can be differentiated down multiple cellular lineages, providing the opportunity to study gene function in multiple different cell types, with a defined genetic background. In addition, as hIPSCs are self-renewing, the starting material is an unlimited resource. Furthermore, many research groups have shown that hIPSCs can be relatively easily genetically manipulated using tools such as zinc finger nucleases, TALENs, and CRISPR-Cas9 systems (52). Here, we show that iPSCs can be differentiated into DCs expressing markers of human CD141^+^ myeloid DCs. However, the levels of the specific lineage markers for CD141^+^ DCs, CLEC9A and XCR1, were quite low in our DC populations, which has been previously described (29). The complex environment and array of signals to which DC progenitors are exposed during development present a challenge to replication *in vitro*. However, a fundamental understanding of human DC development is expanding, and in the future, knowledge of detailed changes in the transcriptional profiles of these cells during development can be applied to help refine differentiation protocols. However, the iPS-DCs described herein expressed multiple DC lineage markers, suggesting that our differentiation protocol is sufficient to derive DC-like cells. In addition, we demonstrated that after differentiation into iPSDMs, IRF5^−/−^ cells are also deficient in IL-6 and TNF-α production, demonstrating for the first time that virus-induced immune responses, including cytokine secretion, can be investigated in iPS-derived cells of multiple myeloid lineages that contain biallelic mutations, thus demonstrating the flexibility of iPSCs as tools to study immune responses to pathogens in multiple cell types.

In two independent studies, response expression quantitative trait loci (reQTLs) were found in IRF5 after stimulation of cells with virus or TLR ligands (53, 54), suggesting that variation within the IRF5 locus may be important in driving differences in expression. It would be interesting in future studies to see if single-nucleotide polymorphisms (SNPs) which drive higher IRF5 expression in human macrophages and DCs also correlate with a heightened inflammatory response to viruses such as IAV, as our data suggest that such individuals may be preferentially susceptible to influenza virus pathogenesis and imply that targeting high IRF5 levels in these individuals could reduce inflammation without impacting virus control. As well as being a useful tool to knock out genes to assess cell-specific function as we have shown here, iPS-DCs could also be used as a tool to explore how common human genetic variants are associated with immune cell responses to various pathogens.

## MATERIALS AND METHODS

### Mice and viral infections.

*Irf5^−/−^* mice were bred in-house on a C57BL/6 background, and their generation has been described previously (12). C57BL/6 WT mice were purchased from Charles River or Envigo. Age- and sex-matched mice between 7 and 12 weeks of age were used in the experiments. Mice were infected intranasally with 3 × 10^3^ PFU A/X-31 influenza virus in 50 μl of sterile phosphate-buffered saline (PBS). Mouse weights were recorded daily, and mice were further monitored for signs of illness.

### Plaque assays.

Influenza virus from the lungs of WT and *Irf5^−/−^* mice was quantified on Madin-Darby canine kidney (MDCK) cell monolayers after a 5-h incubation at 37°C. Cell layers were then overlaid with methylcellulose (Sigma-Aldrich) and incubated at 37°C for a further 48 h. The medium was then removed, and cell layers were washed, fixed, blocked, and further incubated with anti-influenza A nucleoprotein (clone AA5H; Serotec) and then with anti-mouse IgG-horseradish peroxidase (IgG-HRP; Bio-Rad). Plaques were developed using 3-amino-9-ethylcarbazole (AEC) peroxidase substrate solution and subsequently counted via light microscopy.

### Leukocyte isolation, intracellular cytokine staining, and flow cytometry.

BAL fluid and lungs were collected from *Irf5^−/−^* and WT mice at days 2, 4, and 7 p.i. Lung digestions were performed by incubation with collagenase solution (RPMI supplemented with 5% fetal bovine serum [FBS], 1 mg/ml collagenase D [Roche], 5 mM CaCl_2_, 50 mg/ml DNase I [Sigma-Aldrich]), and single-cell suspensions were generated by passing through 100-μm filters. Cells were stained with Zombie Aqua fixable dye, incubated with anti-mouse CD16/CD32 (both BioLegend), and stained for surface markers with a combination of the following antibodies. For murine myeloid panels, cells were stained for surface markers using anti-mouse CD11b-fluorescein isothiocyanate (CD11b-FITC) (M1/70; BioLegend), Ly6C-PerCP/Cy5.5 (HK1.4; BioLegend), SiglecF-PeVio770 (ES22-10D8; Miltenyi Biotec), CD64-Pe/Dazzle (X54-5/7.1; BioLegend), CD45R/B220-APC/Cy7 (RA3-6B2; BioLegend), MHC-II-BV711 (M5/114.15.2; BioLegend), CD11c-BV605 (N418; BioLegend), and SiglecH-Pacific Blue (551; BioLegend). Following surface staining, some cells were fixed and permeabilized with fixation/permeabilization solution (BD Biosciences) and stained with anti-IL-6-phycoerythrin (anti-IL-6-PE) (MP5-20F3; BioLegend) and TNF-α-allophycocyanin (TNF-α-APC) (MP6-XT22; BioLegend). All data were acquired using an Attune NxT flow cytometer (Thermo Fisher Scientific). Electronic compensation was performed with antibody (Ab) capture beads stained separately with individual monoclonal antibodies (MAbs) used in the experimental panel. Data were analyzed using the FlowJo software (TreeStar, Inc.). The total numbers of different cell populations were calculated by multiplying the total number of viable leukocytes (assessed by trypan blue exclusion) by the percentage of positive cells, as detected by flow cytometry.

### Mass cytometry staining for IRF5 expression.

Para-tumor lung tissue samples from metastatic cancer or fibrosis patients were extracted and deemed to show no visible signs of inflammation as assessed by a pathologist. Peripheral blood mononuclear cells (PBMCs) from one donor were run with each lung sample to control for interrun variability. One hundred milliliters of heparinized blood was drawn from a healthy control donor, PBMCs were isolated, and aliquots were frozen until use. Directly conjugated antibodies (CD45-89Y, clone HI30; EpCAM-141Pr, clone 9C4; CD31-151Eu, clone EPR3094; CD68-159Tb, clone KP1; Siglec8-164Dy, clone 7C9) were all purchased from Fluidigm. Conjugations for other antibodies (CD11b-142Nd, clone ICRF44; CD4-145Nd, clone RPA-T4; CD20-147Sm, clone 2H7; CD115-152Sm, clone 12-3A3-1810; CD123-155Gd, clone 6H6; CD14-160Gd, clone M5P2; CD56-166Er, clone NCM/HCD56; CD8-168Er, clone SK1; HLA-DR-169Tm, clone L243; CD3-170Er, clone UCHT1; CD1c-171Yb, clone L161; and CD141-173Yb, clone M80) were performed with the Maxpar X8 multimetal labeling kit (Fluidigm), according to the manufacturer’s instructions.

Cells resuspended at 1 × 10^7^ cells/ml were stained with 5 mmol/liter cisplatin (live/dead; Fluidigm) and surface antibody cocktail before permeabilization with Maxpar nuclear antigen staining buffer and staining with anti-IRF5 (conjugate, 175Lu; clone, EPR17067). An unpermeabilized control was treated with cell staining buffer and stained with intracellular antibodies. Cells were stained with 125 nM Ir-Intercalator according to the manufacturer’s protocols (Fluidigm) and fixed with 1.6% formaldehyde. Cells were counted on a BD Accuri C6 cytometer and resuspended at 2 × 10^6^ cells/ml in 0.1× EQ four-element calibration beads (Fluidigm). Cells were acquired using a CyTOF Helios cytometer (Fluidigm). Data were processed and normalized using the CyTOF software v6.7 (Fluidigm). Data were analyzed using FlowJo (TreeStar, Inc.).

### Mass cytometry analysis.

CyTOF data were analyzed using FlowJo 10.5.2 (TreeStar, Inc.). After gating on live, intact, singlet cells, CD45 versus EpCAM expression was used to identify epithelial cells (CD45^−^ EpCAM^+^) or immune cells (CD45^+^ EpCAM^−^). CD45^+^ EpCAM^−^ cells were downsampled to a maximum of 200,000 per sample, with stained and control samples concatenated into one file. The concatenated file was run through a uniform manifold approximation and projection (UMAP) analysis of the surface markers CD11b, CD4, CD20, CD123, CD68, CD14, Siglec8, CD56, CD8, HLA-DR, CD3, CD1c, CD141, and CD16. Post-UMAP analysis, distinct cell subsets were identified by mapping the expression of specific subset markers back onto the UMAP to define populations of immune cells. Subsets were defined as follows: CD4^+^ T cells, CD3^+^ CD4^+^ CD20^−^; CD8^+^ T cells, CD3^+^ CD20^−^ CD8^+^; B cells, CD3^−^ CD20^+^; NK cells, CD3^−^ CD20^−^ CD56^+^; CD14^+^ monocytes, CD16^−^ CD11b^+^ CD14^+^ HLA-DR^+^; CD16^+^ monocytes, CD14^−^ CD11b^+^ CD16^+^ HLA-DR^+^; macrophages, CD11b^+^ CD68^+^ HLA-DR^+^; pDCs, CD123^+^ CD11b^+^ HLA-DR^+^; CD141^+^ cDCs, CD11b^+^ HLA-DR^+^ CD1c^−^ CD141^+^; CD1c^+^ cDCs, CD11b^+^ HLA-DR^+^ CD1c^+^ CD141^−^; eosinophils, Siglec8^+^ CD123^−^; basophils, Siglec8^+^ CD123^+^; and epithelial cells, CD45^−^ EpCAM^+^. Individual samples were identified by gating on event length versus sample identifier (ID), and the median value was determined for IRF5 for each individual sample.

### Generation of blood-derived human dendritic cells.

PBMCs from three independent donors were isolated from leukapheresis products using Lymphoprep density gradient centrifugation and SepMate PBMC isolation tubes (StemCell Technologies), under the Weatherall Institute of Molecular Medicine, University of Oxford Human Tissue Authority license 12433. Human CD14 microbeads were used in combination with LS columns (both Miltenyi Biotec) to positively select CD14^+^ blood monocytes. CD14^+^ cells were seeded at a density of 3 × 10^6^ to 5 × 10^6^ isolated monocytes in 3 ml of RPMI medium supplemented with 10% heat-inactivated fetal bovine serum (FBS; Sigma-Aldrich), 250 IU/ml IL-4, and 800 IU/ml GM-CSF into a 6-well plate and incubated at 37°C for 2 days. After 2 days, 1.5 ml of medium was removed from each well, and 1.5 ml of fresh medium supplemented with 500 IU/ml IL-4 and 1,600 IU/ml GM-CSF was added. After a further 3-day incubation, cells were harvested at the immature phenotype and assayed or further matured with LPS at 10 μg/ml for 24 h.

### hIPSCs.

The healthy control hIPSC line Kolf2 was acquired through the Human Induced Pluripotent Stem Cells Initiative Consortium (HipSci; http://www.hipsci.org/), through which it was also characterized (55). The generation of IRF5^−/−^ iPSCs has been previously described (16). Briefly, Kolf2 iPSCs were dissociated to single cells and nucleofected (Amaxa2b nucleofector; Lonza) with Cas9 coding plasmid (hCas9; Addgene 41815), single guide RNA (sgRNA) plasmid (CRISPR guides, left CRISPR_IRF5, CCAAGTGGAAGGCCAACCTGCGC, and right CRISPR_IRF5, GACTTCCGCCTCATCTACGACGG), and donor plasmid containing 5′ and 3′ homology arms for IRF5 targeting and the pL1-EF1αPuro-L2 cassette. Postnucleofection, cells were selected for up to 11 days with 0.25 μg/ml puromycin, after which individual colonies were picked onto 96-well plates, grown to confluence, and then replica plated. To genotype individual clones from 96-well replica plates, cells were lysed and used for PCR amplification with LongAmp *Taq* DNA polymerase (NEB). Insertion of the cassette into the correct locus was confirmed by visualizing on 1% E-gel (Life Technologies). PCR products were generated by gene-specific and cassette-specific primers, with single integration of cassette confirmed by a quantitative PCR (qPCR) copy number assay. Positive clones were then screened for damage to the nontargeted allele via PCR and Sanger sequencing. To generate our complemented IRF5 iPSC line (IRF5^Comp^) and restore the expression of functional IRF5 in the IRF5^−/−^ iPSCs, we generated the AAVS1 EF1a-IRF5-PGK-puro targeting vector by Gibson Assembly. The Gibson Assembly product was transformed into One Shot TOP10 chemically competent Escherichia coli cells (Thermo Fisher Scientific), and positive colonies were picked. Isolated plasmids from the positive colonies were taken to confirm the presence and sequence of elongation factor 1 alpha (EF1α)-IRF5 in the targeting vector by restriction digestions, PCR, and sequencing. Subsequently, the targeting vector was transformed into competent E. coli cells to isolate endotoxin-free plasmids to transform into the IRF5^−/−^ iPSCs. We transfected the mutant human hIPSCs with TALEN-L (CCCCTCCACCCCACAGT), TALEN-R (AGGATTGGTGACAGAAA), and targeting vector via nucleofection (Amaxa Biosystems). The resultant targeted cells were selected on puromycin for 7 days, and the surviving colonies were picked and expanded. The positive clones were confirmed by reverse transcription-quantitative PCR (RT-qPCR) for *IRF5* expression and flow cytometry for protein expression. Prior to differentiation, iPSCs were grown feeder free using the Essential 8 Flex medium kit (Thermo Fisher Scientific) on vitronectin (VTN-N; Thermo Fisher Scientific)-coated plates, as per the manufacturer’s instructions, to 70 to 80% confluence. iPSCs were harvested for differentiation using Versene solution (Thermo Fisher Scientific).

### Differentiation of iPSCs to dendritic cells and macrophages.

To differentiate iPSCs to dendritic cells, slight modifications were made to a previously published protocol (29). Briefly, upon reaching confluence, iPSCs were harvested and plated into Essential 8 Flex medium supplemented with 50 ng/ml bone morphogenetic protein 4 (BMP-4; Bio-Techne), 20 ng/ml stem cell factor (SCF; Bio-Techne), 50 ng/ml vascular endothelial growth factor (VEGF; Peprotech EC Ltd.), and 50 ng/ml GM-CSF (Peprotech EC Ltd.) in ultralow attachment (ULA) plates (Corning). The medium was changed to X-VIVO-15 (Lonza), with sequential removal of BMP-4 by day 5, VEGF by approximately day 14, and SCF by approximately day 19. In addition, IL-4 (Peprotech EC Ltd.) was added sequentially in increasing concentrations, starting from approximately day 12 at 25 ng/ml and increasing to 100 ng/ml by approximately day 20. By day 20, floating immature DCs were harvested from ULA plates, filtered through 70-μm filters (Corning), counted, and seeded at 1 × 10^6^ per well of 6-well CellBind plates (Corning) in X-VIVO-15 medium supplemented with 100 ng/ml IL-4 and 50 ng/ml GM-CSF. iPS-DCs were used for assays at the immature phase between 4 and 5 days postseeding in CellBind plates. In addition, iPS-DCs could be matured for a further 48 h at 5 days postplating by the addition of 50 ng/ml GM-CSF, 100 ng/ml IL-4, 20 ng/ml IFN-γ (Bio-Techne), 50 ng/ml TNF-α (Bio-Techne), 10 ng/ml IL-1β (Bio-Techne), and 1 μg/ml prostaglandin E2 (PGE_2_; Sigma-Aldrich) to induce further expression of CD141^+^ DC lineage markers. For the assays, floating iPS-DCs were harvested from differentiation plates, washed with PBS, counted, and seeded in X-VIVO-15 medium without cytokines at an assay-dependent concentration. To differentiate iPSCs to macrophages, the approaches of Hale et al. (22) and van Wilgenburg et al. (56) were modified to allow for feeder-free differentiation. Briefly, upon reaching confluence, human iPSCs were collected and transferred into Essential 8 Flex medium supplemented with 50 ng/ml BMP-4 (Bio-Techne), 20 ng/ml SCF (Bio-Techne), and 50 ng/ml VEGF (Peprotech EC Ltd.) in ultralow attachment plates (Corning) for 4 days to generate embryoid bodies (EBs). On day 5, EBs were used for the generation of myeloid precursor cells by plating into 6-well tissue culture-treated plates (Corning) coated for 2 h at room temperature with 0.1% gelatin in X-VIVO-15 medium supplemented with 25 ng/ml IL-3 (Bio-Techne) and 50 ng/ml M-CSF (Bio-Techne). After several weeks, floating myeloid precursors were harvested and terminally differentiated into matured macrophages (iPSDMs) in the presence of higher concentrations of macrophage colony-stimulating factor (M-CSF; 100 ng/ml) for 7 days. For the experiments, macrophages were detached using Lidocaine solution (4 mg/ml lidocaine-HCl with 10 mM EDTA in PBS) and seeded at 2 × 10^5^ cells per well (24-well plate) or 1 × 10^6^ cells per well (six-well plate).

### Preparation of RNA and RT-qPCR.

iPS-DCs were harvested from the plates, and RNA was prepared using the RNeasy minikit (Qiagen). RNA was reverse transcribed with the QuantiTect reverse transcription (RT) kit (Qiagen), according to the manufacturer’s protocol. All RT-qPCR experiments were performed with TaqMan gene expression assays and TaqMan gene expression master mix (Applied Biosystems) on the Applied Biosystems StepOne real-time PCR system. RT-qPCR data were analyzed via the comparative threshold cycle (*C_T_*) method with glyceraldehyde 3-phosphate dehydrogenase (GAPDH) as an endogenous control.

### Flow cytometric analysis of iPS-DCs.

For analysis of surface markers on iPS-DCs, cells were stained with Zombie Aqua fixable dye (BioLegend), Fc receptors were blocked using human TruStain FcX (BioLegend), and cells were then subsequently stained for surface markers with a combination of the following antibodies: anti-human HLA-DR-Alexa Fluor 488 (AF488) (L243; BioLegend) or CD14-FITC (M5E2; BioLegend), CD83-PerCP/Cy5.5 (HB15e; BioLegend) or CD1c-PerCP/Cy5.5 (L161; BioLegend), CD141-PE/Cy7 (M80; BioLegend) or DC-SIGN-PE/Cy7 (9E9A8; BioLegend), or XCR1-PE (FAB8571; Bio-Techne), CD11c-APC/Cy7 (Bu15; BioLegend), CLEC9A-APC (8F9; BioLegend), CD86-BV711 (IT2.2; BioLegend), CD303-BV785 (201A; BioLegend) or HLA-DR-BV785 (L243; BioLegend), and HLA-A,B,C-Pacific Blue (W6/32; BioLegend). For the detection of IRF5 or IAV nucleoprotein, cells were stained with Zombie Aqua fixable dye, fixed with 4% paraformaldehyde, and permeabilized with 0.5% Triton X-100, followed by incubation with human TruStain FcX and staining with IRF5-AF647 (EPR6094; Abcam) or anti-influenza A virus nucleoprotein antibody (431; Abcam) in 0.1% Triton X-100 solution. All data were acquired using an LSRFortessa flow cytometer (BD Biosciences). Electronic compensation was performed with Ab capture beads stained separately with individual MAbs used in the experimental panel. Data were analyzed using the FlowJo software (TreeStar, Inc.).

### Infection of iPS-DCs and iPSDMs with IAV.

iPS-DCs, iPSDMs, or human monocyte-derived DCs (mDCs) were infected with A/X-31 influenza virus at a multiplicity of infection (MOI) of 1 by the addition of virus to culture supernatant and centrifugation at 630 × *g* for 20 min at room temperature, after which the medium was replaced with fresh culture medium.

### Immunostaining for confocal microscopy.

iPS-DCs were harvested from plates and spun onto slides coated with 0.01% poly-l-lysine using a Cytospin cytocentrifuge. Samples were blocked and permeabilized in 2% Triton X-100 (Sigma-Aldrich) in 5% FBS diluted in PBS. Primary antibodies were applied at room temperature in 0.25% Triton X-100 in 5% FBS diluted in PBS for 1 h and then rinsed 3 times with PBS. Secondary antibodies were applied in the same manner. Nuclei were counterstained with 10 nM DAPI (4′,6-diamidino-2-phenylindole) dilactate diluted in PBS for 30 min, and samples were rinsed 6 times with PBS, mounted in ProLong Gold reagent with added DAPI (Invitrogen), and analyzed using a Leica SP8 digital light sheet (DLS) microscope.

### TLR/RIG-I stimulations.

iPS-DCs were plated at 1 × 10^4^ cells per well in 200 μl of X-VIVO-15 medium without cytokines. TLR ligands were added directly to the medium, and supernatants were harvested after a 24-h incubation at 37°C. For the assays, TLR ligands were used at the following concentrations: Pam3CSK4, 300 ng/ml (InvivoGen); poly(I·C), 50 μg/ml (InvivoGen); lipopolysaccharide, 500 ng/ml (Sigma-Aldrich); imiquimod, 50 μg/ml (InvivoGen); and ODN 2216, 3 μg/ml (Miltenyi Biotech). For RIG-I stimulation, 1 μg of 3p-hpRNA was complexed with LyoVec (InvivoGen) for 15 min at room temperature and then added to iPS-DCs at 10 ng/ml.

### Cytokine and chemokine analysis.

Human IL-6 and TNF-α protein were measured by an enzyme-linked immunosorbent assay (ELISA) (BioLegend). ELISAs for human IFN-α and IFN-β were performed on supernatants harvested from mock-infected and IAV-infected iPS-DCs using the VeriKine human interferon alpha/interferon beta ELISA kit (PBL Assay Science). Murine BAL fluid cytokines were detected using the LEGENDPlex mouse inflammation panel (13-plex; BioLegend), as per the manufacturer’s instructions, at 2, 4, and 6 days p.i. and analyzed using the LEGENDPlex analysis software. ELISAs for mouse IFN-α and IFN-β were performed on BAL fluid from naive mice and mice at 2 days p.i. using VeriKine mouse interferon alpha/interferon beta ELISA kits (PBL Assay Science).

### Blocking assays.

For blocking assays, cells were either preincubated for 1 h with 5 μg/ml inhibitor (IMD 0354, IKKβ inhibitor; Santa Cruz Biotechnology), or inhibitor was added directly with 5 μM viral inoculum (TLR7 inhibitor, ODN 20958; Miltenyi Biotech). For type I IFN blocking, cells were preincubated with 5 μg/ml anti-IFNAR1 antibody (Sigma-Aldrich) for 1 h prior to infection with A/X-31 influenza virus without removal of antibody.

### Statistical analysis.

Statistical significance was performed using the GraphPad Prism software. The Mann-Whitney U or Student’s *t* test was used for two-group comparisons. For comparison of IRF5 expression levels between lung cell subsets identified via CyTOF, a repeated-measures one-way analysis of variance (ANOVA) was used. A *P* value of ≤0.05 was considered to be significant. For all tests performed, *P* values are reported as follows: n.s., >0.05; *, ≤0.05; **, ≤0.01; ***, ≤0.001; and ****, ≤0.0001.

### Ethics statement.

All animal studies were performed at Cardiff University (Heath Park research support facility) under UK Home Office Project License number P7867DADD, as approved by the UK Home Office, London, United Kingdom. Written consent was obtained for the use of cell lines for the HipSci project from healthy volunteers. A favorable ethical opinion was granted by the National Research Ethics Service (NRES) Research Ethics Committee Yorkshire and The Humber-Leeds West, under reference number 15/YH/0391. Lung tissue samples were obtained from lung cancer and fibrosis patients from Oxford Radcliffe Biobank with written consent; a favorable ethical opinion was granted by the South Central-Oxford C Research Ethics Committee for the collection and frozen storage of both tumor and para-tumor lung samples (reference number 09/H0606/5 + 5).

## References

[1] de Jong MD, Simmons CP, Thanh TT, Hien VM, Smith GJD, Chau TNB, Hoang DM, Van Vinh Chau N, Khanh TH, Dong VC, Qui PT, Van Cam B, Ha DQ, Guan Y, Peiris JSM, Chinh NT, Hien TT, Farrar J 2006 Fatal outcome of human influenza A (H5N1) is associated with high viral load and hypercytokinemia. Nat Med 12:1203–1207. doi:10.1038/nm1477. 16964257 PMC4333202

[2] Humphreys IR, Walzl G, Edwards L, Rae A, Hill S, Hussell T 2003 A critical role for OX40 in T cell-mediated immunopathology during lung viral infection. J Exp Med 198:1237–1242. doi:10.1084/jem.20030351. 14568982 PMC2194232

[3] GeurtsvanKessel CH, Willart MAM, van Rijt LS, Muskens F, Kool M, Baas C, Thielemans K, Bennett C, Clausen BE, Hoogsteden HC, Osterhaus A, Rimmelzwaan GF, Lambrecht BN 2008 Clearance of influenza virus from the lung depends on migratory langerin^+^ CD11b^−^ but not plasmacytoid dendritic cells. J Exp Med 205:1621–1634. doi:10.1084/jem.20071365. 18591406 PMC2442640

[4] Liu J, Cao X 2016 Cellular and molecular regulation of innate inflammatory responses. Cell Mol Immunol 13:711–721. doi:10.1038/cmi.2016.58. 27818489 PMC5101451

[5] Iwasaki A, Pillai PS 2014 Innate immunity to influenza virus infection. Nat Rev Immunol 14:315–328. doi:10.1038/nri3665. 24762827 PMC4104278

[6] Tavares LP, Teixeira MM, Garcia CC 2017 The inflammatory response triggered by influenza virus: a two edged sword. Inflamm Res 66:283–302. doi:10.1007/s00011-016-0996-0. 27744631

[7] Khoyratty TE, Udalova IA 2018 Diverse mechanisms of IRF5 action in inflammatory responses. Int J Biochem Cell Biol 99:38–42. doi:10.1016/j.biocel.2018.03.012. 29578052

[8] Krausgruber T, Saliba D, Ryzhakov G, Lanfrancotti A, Blazek K, Udalova IA 2010 IRF5 is required for late-phase TNF secretion by human dendritic cells. Blood 115:4421–4430. doi:10.1182/blood-2010-01-263020. 20237317

[9] Douagi I, McInerney GM, Hidmark AS, Miriallis V, Johansen K, Svensson L, Karlsson Hedestam GB 2007 Role of interferon regulatory factor 3 in type I interferon responses in rotavirus-infected dendritic cells and fibroblasts. J Virol 81:2758–2768. doi:10.1128/JVI.01555-06. 17215281 PMC1865971

[10] Honda K, Yanai H, Negishi H, Asagiri M, Sato M, Mizutani T, Shimada N, Ohba Y, Takaoka A, Yoshida N, Taniguchi T 2005 IRF-7 is the master regulator of type-I interferon-dependent immune responses. Nature 434:772–777. doi:10.1038/nature03464. 15800576

[11] Ren J, Chen X, Chen ZJ 2014 IKKβ is an IRF5 kinase that instigates inflammation. Proc Natl Acad Sci U S A 111:17438–17443. doi:10.1073/pnas.1418516111. 25326420 PMC4267374

[12] Takaoka A, Yanai H, Kondo S, Duncan G, Negishi H, Mizutani T, Kano S, Honda K, Ohba Y, Mak TW, Taniguchi T 2005 Integral role of IRF-5 in the gene induction programme activated by Toll-like receptors. Nature 434:243–249. doi:10.1038/nature03308. 15665823

[13] Eames HL, Corbin AL, Udalova IA 2016 Interferon regulatory factor 5 in human autoimmunity and murine models of autoimmune disease. Transl Res 167:167–182. doi:10.1016/j.trsl.2015.06.018. 26207886

[14] Thackray LB, Shrestha B, Richner JM, Miner JJ, Pinto AK, Lazear HM, Gale M, Diamond MS 2014 Interferon regulatory factor 5-dependent immune responses in the draining lymph node protect against West Nile virus infection. J Virol 88:11007–11021. doi:10.1128/JVI.01545-14. 25031348 PMC4178807

[15] Paun A, Bankoti R, Joshi T, Pitha PM, Stäger S 2011 Critical role of IRF-5 in the development of T helper 1 responses to Leishmania donovani infection. PLoS Pathog 7:e1001246. doi:10.1371/journal.ppat.1001246. 21253574 PMC3017120

[16] Yeung ATY, Hale C, Lee AH, Gill EE, Bushell W, Parry-Smith D, Goulding D, Pickard D, Roumeliotis T, Choudhary J, Thomson N, Skarnes WC, Dougan G, Hancock R 2017 Exploiting induced pluripotent stem cell-derived macrophages to unravel host factors influencing Chlamydia trachomatis pathogenesis. Nat Commun 8:15013. doi:10.1038/ncomms15013. 28440293 PMC5414054

[17] Yanai H, Chen H.-m, Inuzuka T, Kondo S, Mak TW, Takaoka A, Honda K, Taniguchi T 2007 Role of IFN regulatory factor 5 transcription factor in antiviral immunity and tumor suppression. Proc Natl Acad Sci U S A 104:3402–3407. doi:10.1073/pnas.0611559104. 17360658 PMC1805533

[18] Mancl ME, Hu G, Sangster-Guity N, Olshalsky SL, Hoops K, Fitzgerald-Bocarsly P, Pitha PM, Pinder K, Barnes BJ 2005 Two discrete promoters regulate the alternatively spliced human interferon regulatory factor-5 isoforms. J Biol Chem 280:21078–21090. doi:10.1074/jbc.M500543200. 15805103

[19] Lin KL, Suzuki Y, Nakano H, Ramsburg E, Gunn MD 2008 CCR2^+^ monocyte-derived dendritic cells and exudate macrophages produce influenza-induced pulmonary immune pathology and mortality. J Immunol 180:2562–2572. doi:10.4049/jimmunol.180.4.2562. 18250467

[20] Ellis GT, Davidson S, Crotta S, Branzk N, Papayannopoulos V, Wack A 2015 TRAIL^+^ monocytes and monocyte‐related cells cause lung damage and thereby increase susceptibility to influenza-Streptococcus pneumoniae coinfection. EMBO Rep 16:1203–1218. doi:10.15252/embr.201540473. 26265006 PMC4576987

[21] Osugi Y, Vuckovic S, Hart D 2002 Myeloid blood CD11c^+^ dendritic cells and monocyte-derived dendritic cells differ in their ability to stimulate T lymphocytes. Blood 100:2858–2866. doi:10.1182/blood.V100.8.2858. 12351396

[22] Hale C, Yeung A, Goulding D, Pickard D, Alasoo K, Powrie F, Dougan G, Mukhopadhyay S 2015 Induced pluripotent stem cell derived macrophages as a cellular system to study Salmonella and other pathogens. PLoS One 10:e0124307. doi:10.1371/journal.pone.0124307. 25946027 PMC4422593

[23] Hussell T, Pennycook A, Openshaw P 2001 Inhibition of tumor necrosis factor reduces the severity of virus-specific lung immunopathology. Eur J Immunol 31:2566–2573. doi:10.1002/1521-4141(200109)31:9<2566::AID-IMMU2566>3.0.CO;2-L. 11536154

[24] Vlahos R, Stambas J, Bozinovski S, Broughton BRS, Drummond GR, Selemidis S 2011 Inhibition of Nox2 oxidase activity ameliorates influenza A virus-induced lung inflammation. PLoS Pathog 7:e1001271. doi:10.1371/journal.ppat.1001271. 21304882 PMC3033375

[25] Chen X, Zhou L, Peng N, Yu H, Li M, Cao Z, Lin Y, Wang X, Li Q, Wang J, She Y, Zhu C, Lu M, Zhu Y, Liu S 2017 MicroRNA-302a suppresses influenza A virus-stimulated interferon regulatory factor-5 expression and cytokine storm induction. J Biol Chem 292:21291–21303. doi:10.1074/jbc.M117.805937. 29046356 PMC5766960

[26] Davidson S, Crotta S, McCabe TM, Wack A 2014 Pathogenic potential of interferon αβ in acute influenza infection. Nat Commun 5:3864. doi:10.1038/ncomms4864. 24844667 PMC4033792

[27] Xu L, Yoon H, Zhao MQ, Liu J, Ramana CV, Enelow RI 2004 Cutting edge: pulmonary immunopathology mediated by antigen-specific expression of TNF-α by antiviral CD8^+^ T cells. J Immunol 173:721–725. doi:10.4049/jimmunol.173.2.721. 15240656

[28] Song M-S, Cho Y-H, Park S-J, Pascua PNQ, Baek YH, Kwon H-I, Lee O-J, Kong B-W, Kim H, Shin E-C, Kim C-J, Choi YK 2013 Early regulation of viral infection reduces inflammation and rescues Mx-positive mice from lethal avian influenza infection. Am J Pathol 182:1308–1321. doi:10.1016/j.ajpath.2012.12.022. 23395090

[29] Sachamitr P, Leishman AJ, Davies TJ, Fairchild PJ 2018 Directed differentiation of human induced pluripotent stem cells into dendritic cells displaying tolerogenic properties and resembling the CD141^+^ subset. Front Immunol 8:1935. doi:10.3389/fimmu.2017.01935. 29358940 PMC5766641

[30] Forbester JL, Lees EA, Goulding D, Forrest S, Yeung A, Speak A, Clare S, Coomber EL, Mukhopadhyay S, Kraiczy J, Schreiber F, Lawley TD, Hancock REW, Uhlig HH, Zilbauer M, Powrie F, Dougan G 2018 Interleukin-22 promotes phagolysosomal fusion to induce protection against Salmonella enterica Typhimurium in human epithelial cells. Proc Natl Acad Sci U S A 115:10118–10123. doi:10.1073/pnas.1811866115. 30217896 PMC6176607

[31] Mukhopadhyay S, Heinz E, Porreca I, Alasoo K, Yeung A, Yang H-T, Schwerd T, Forbester JL, Hale C, Agu CA, Choi YH, Rodrigues J, Capitani M, Jostins-Dean L, Thomas DC, Travis S, Gaffney D, Skarnes WC, Thomson N, Uhlig HH, Dougan G, Powrie F 2020 Loss of IL-10 signaling in macrophages limits bacterial killing driven by prostaglandin E2. J Exp Med 217:e20180649. doi:10.1084/jem.20180649. 31819956 PMC7041704

[32] Collin M, Bigley V 2018 Human dendritic cell subsets: an update. Immunology 154:3–20. doi:10.1111/imm.12888. 29313948 PMC5904714

[33] Colletti NJ, Liu H, Gower AC, Alekseyev YO, Arendt CW, Shaw MH 2016 TLR3 signaling promotes the induction of unique human BDCA-3 dendritic cell populations. Front Immunol 7:88. doi:10.3389/fimmu.2016.00088. 27014268 PMC4789364

[34] Breton G, Zheng S, Valieris R, Tojal da Silva I, Satija R, Nussenzweig MC 2016 Human dendritic cells (DCs) are derived from distinct circulating precursors that are precommitted to become CD1c^+^ or CD141^+^ DCs. J Exp Med 213:2861–2870. doi:10.1084/jem.20161135. 27864467 PMC5154947

[35] Lauder SN, Jones E, Smart K, Bloom A, Williams AS, Hindley JP, Ondondo B, Taylor PR, Clement M, Fielding C, Godkin AJ, Jones SA, Gallimore AM 2013 Interleukin-6 limits influenza-induced inflammation and protects against fatal lung pathology. Eur J Immunol 43:2613–2625. doi:10.1002/eji.201243018. 23857287 PMC3886386

[36] Dienz O, Rud JG, Eaton SM, Lanthier PA, Burg E, Drew A, Bunn J, Suratt BT, Haynes L, Rincon M 2012 Essential role of IL-6 in protection against H1N1 influenza virus by promoting neutrophil survival in the lung. Mucosal Immunol 5:258–266. doi:10.1038/mi.2012.2. 22294047 PMC3328598

[37] Kaiser L, Fritz RS, Straus SE, Gubareva L, Hayden FG 2001 Symptom pathogenesis during acute influenza: interleukin-6 and other cytokine responses. J Med Virol 64:262–268. doi:10.1002/jmv.1045. 11424113

[38] Hayden FG, Fritz R, Lobo MC, Alvord W, Strober W, Straus SE 1998 Local and systemic cytokine responses during experimental human influenza A virus infection. Relation to symptom formation and host defense. J Clin Invest 101:643–649. doi:10.1172/JCI1355. 9449698 PMC508608

[39] Lopez-Pelaez M, Lamont DJ, Peggie M, Shpiro N, Gray NS, Cohen P 2014 Protein kinase IKKβ-catalyzed phosphorylation of IRF5 at Ser462 induces its dimerization and nuclear translocation in myeloid cells. Proc Natl Acad Sci U S A 111:17432–17437. doi:10.1073/pnas.1418399111. 25326418 PMC4267347

[40] Pang IK, Pillai PS, Iwasaki A 2013 Efficient influenza A virus replication in the respiratory tract requires signals from TLR7 and RIG-I. Proc Natl Acad Sci U S A 110:13910–13915. doi:10.1073/pnas.1303275110. 23918369 PMC3752242

[41] Zhang Z, Yuan B, Lu N, Facchinetti V, Liu Y-J 2011 DHX9 pairs with IPS-1 to sense double-stranded RNA in myeloid dendritic cells. J Immunol 187:4501–4508. doi:10.4049/jimmunol.1101307. 21957149 PMC3656476

[42] Kandasamy M, Suryawanshi A, Tundup S, Perez JT, Schmolke M, Manicassamy S, Manicassamy B 2016 RIG-I signaling is critical for efficient polyfunctional T cell responses during influenza virus infection. PLoS Pathog 12:e1005754. doi:10.1371/journal.ppat.1005754. 27438481 PMC4954706

[43] Schoenemeyer A, Barnes BJ, Mancl ME, Latz E, Goutagny N, Pitha PM, Fitzgerald KA, Golenbock DT 2005 The interferon regulatory factor, IRF5, is a central mediator of Toll-like receptor 7 signaling. J Biol Chem 280:17005–17012. doi:10.1074/jbc.M412584200. 15695821

[44] Silvin A, Yu CI, Lahaye X, Imperatore F, Brault J-B, Cardinaud S, Becker C, Kwan W-H, Conrad C, Maurin M, Goudot C, Marques-Ladeira S, Wang Y, Pascual V, Anguiano E, Albrecht RA, Iannacone M, García-Sastre A, Goud B, Dalod M, Moris A, Merad M, Palucka AK, Manel N 2017 Constitutive resistance to viral infection in human CD141^+^ dendritic cells. Sci Immunol 2:eaai8071. doi:10.1126/sciimmunol.aai8071. 28783704 PMC5749640

[45] Lazear HM, Lancaster A, Wilkins C, Suthar MS, Huang A, Vick SC, Clepper L, Thackray L, Brassil MM, Virgin HW, Nikolich-Zugich J, Moses AV, Gale M, Früh K, Diamond MS 2013 IRF-3, IRF-5, and IRF-7 coordinately regulate the type I IFN response in myeloid dendritic cells downstream of MAVS signaling. PLoS Pathog 9:e1003118. doi:10.1371/journal.ppat.1003118. 23300459 PMC3536698

[46] Purtha WE, Swiecki M, Colonna M, Diamond MS, Bhattacharya D 2012 Spontaneous mutation of the Dock2 gene in Irf5^−/−^ mice complicates interpretation of type I interferon production and antibody responses. Proc Natl Acad Sci U S A 109:E898–E904. doi:10.1073/pnas.1118155109. 22431588 PMC3326475

[47] Andrilenas KK, Ramlall V, Kurland J, Leung B, Harbaugh AG, Siggers T 2018 DNA-binding landscape of IRF3, IRF5 and IRF7 dimers: implications for dimer-specific gene regulation. Nucleic Acids Res 46:2509–2520. doi:10.1093/nar/gky002. 29361124 PMC5861432

[48] Barnes BJ, Kellum MJ, Field AE, Pitha PM 2002 Multiple regulatory domains of IRF-5 control activation, cellular localization, and induction of chemokines that mediate recruitment of T lymphocytes. Mol Cell Biol 22:5721–5740. doi:10.1128/mcb.22.16.5721-5740.2002. 12138184 PMC133975

[49] Hatesuer B, Hoang HTT, Riese P, Trittel S, Gerhauser I, Elbahesh H, Geffers R, Wilk E, Schughart K 2017 Deletion of *Irf3* and *Irf7* genes in mice results in altered interferon pathway activation and granulocyte-dominated inflammatory responses to influenza A infection. J Innate Immun 9:145–161. doi:10.1159/000450705. 27811478 PMC6738875

[50] Ciancanelli MJ, Huang SXL, Luthra P, Garner H, Itan Y, Volpi S, Lafaille FG, Trouillet C, Schmolke M, Albrecht RA, Israelsson E, Lim HK, Casadio M, Hermesh T, Lorenzo L, Leung LW, Pedergnana V, Boisson B, Okada S, Picard C, Ringuier B, Troussier F, Chaussabel D, Abel L, Pellier I, Notarangelo LD, Garcia-Sastre A, Basler CF, Geissmann F, Zhang S-Y, Snoeck H-W, Casanova J-L 2015 Life-threatening influenza and impaired interferon amplification in human IRF7 deficiency. Science 348:448–453. doi:10.1126/science.aaa1578. 25814066 PMC4431581

[51] Mestas J, Hughes C 2004 Of mice and not men: differences between mouse and human immunology. J Immunol 172:2731–2738. doi:10.4049/jimmunol.172.5.2731. 14978070

[52] Hockemeyer D, Jaenisch R 2016 Induced pluripotent stem cells meet genome editing. Cell Stem Cell 18:573–586. doi:10.1016/j.stem.2016.04.013. 27152442 PMC4871596

[53] Lee MN, Ye C, Villani A-C, Raj T, Li W, Eisenhaure TM, Imboywa SH, Chipendo PI, Ran FA, Slowikowski K, Ward LD, Raddassi K, McCabe C, Lee MH, Frohlich IY, Hafler DA, Kellis M, Raychaudhuri S, Zhang F, Stranger BE, Benoist CO, De Jager PL, Regev A, Hacohen N 2014 Common genetic variants modulate pathogen-sensing responses in human dendritic cells. Science 343:1246980. doi:10.1126/science.1246980. 24604203 PMC4124741

[54] Çalışkan M, Baker SW, Gilad Y, Ober C 2015 Host genetic variation influences gene expression response to rhinovirus infection. PLoS Genet 11:e1005111. doi:10.1371/journal.pgen.1005111. 25874939 PMC4395341

[55] Leha A, HipSci Consortium, Moens N, Meleckyte R, Culley OJ, Gervasio MK, Kerz M, Reimer A, Cain SA, Streeter I, Folarin A, Stegle O, Kielty CM, Durbin R, Watt FM, Danovi D 2016 A high-content platform to characterise human induced pluripotent stem cell lines. Methods 96:85–96. doi:10.1016/j.ymeth.2015.11.012. 26608109 PMC4773406

[56] van Wilgenburg B, Browne C, Vowles J, Cowley SA 2013 Efficient, long term production of monocyte-derived macrophages from human pluripotent stem cells under partly-defined and fully-defined conditions. PLoS One 8:e71098. doi:10.1371/journal.pone.0071098. 23951090 PMC3741356

